# Enhanced heavy metal removal from an aqueous environment using an eco-friendly and sustainable adsorbent

**DOI:** 10.1038/s41598-020-73570-7

**Published:** 2020-10-05

**Authors:** Wanqi Zhang, Yuhong An, Shujing Li, Zhechen Liu, Zhangjing Chen, Yukun Ren, Sunguo Wang, Xiaotao Zhang, Ximing Wang

**Affiliations:** 1 College of Material Science and Art Design, Inner Mongolia Agricultural University, Hohhot, China; 2 Department of Sustainable Biomaterials, Virginia Polytechnic Institute and State University, Blacksburg, VA USA; 3 Bioimaging Research, Sanofi Global R&D, Framingham, MA USA; 4 Sungro Bioresource and Bioenergy Technologies Corp, Alberta, Canada; 5 College of Science, Inner Mongolia Agricultural University, Hohhot, China; 6 Inner Mongolia Key Laboratory of Sandy Shrubs Fibrosis and Energy Development and Utilization, Hohhot, China

**Keywords:** Chemical modification, Environmental chemistry

## Abstract

Thiol-lignocellulose sodium bentonite (TLSB) nanocomposites can effectively remove heavy metals from aqueous solutions. TLSB was formed by using –SH group-modified lignocellulose as a raw material, which was intercalated into the interlayers of hierarchical sodium bentonite. Characterization of TLSB was then performed with BET, FTIR, XRD, TGA, PZC, SEM, and TEM analyses. The results indicated that thiol-lignocellulose molecules may have different influences on the physicochemical properties of sodium bentonite, and an intercalated–exfoliated structure was successfully formed. The TLSB nanocomposite was subsequently investigated to validate its adsorption and desorption capacities for the zinc subgroup ions Zn(II), Cd(II) and Hg(II). The optimum adsorption parameters were determined based on the TLSB nanocomposite dosage, concentration of zinc subgroup ions, solution pH, adsorption temperature and adsorption time. The results revealed that the maximum adsorption capacity onto TLSB was 357.29 mg/g for Zn(II), 458.32 mg/g for Cd(II) and 208.12 mg/g for Hg(II). The adsorption kinetics were explained by the pseudo-second-order model, and the adsorption isotherm conformed to the Langmuir model, implying that the dominant chemical adsorption mechanism on TLSB is monolayer coverage. Thermodynamic studies suggested that the adsorption is spontaneous and endothermic. Desorption and regeneration experiments revealed that TLSB could be desorbed with HCl to recover Zn(II) and Cd(II) and with HNO_3_ to recover Hg(II) after several consecutive adsorption/desorption cycles. The adsorption mechanism was investigated through FTIR, EDX and SEM, which demonstrated that the introduction of thiol groups improved the adsorption capacity. All of these results suggested that TLSB is an eco-friendly and sustainable adsorbent for the extraction of Zn(II), Cd(II) and Hg(II) ions in aqueous media.

## Introduction

There is growing concern regarding the environmental contamination of water bodies worldwide and its health effects on mankind^1^. In recent decades, the widespread release and leaching of heavy metals have resulted in contamination of the aqueous environment^2^. According to the United Nations Development Program, only one-third of the world's population will have access to clean water in 2025 based on current water usage trends^3^. Heavy metals such as the zinc subgroup ions Zn(II), Cd(II) and Hg(II) in water will cause great threats to human health because they are hazardous and cancer-causing. Zn(II) is commonly involved in the production of brass, batteries, alloys, powders, paints, metal plating, electroplating, mining and processing, etc.^4^ Zn(II) can react with other chemicals such as acids and oxygen to form potentially toxic compounds, so zinc ions can be detrimental to flora and fauna. Zinc is also responsible for numerous illnesses, including pancreatic damage and anemia, and is nausea-inducing^5^. The Food and Drug Administration (FDA) has established a zinc concentration limit in potable water of 5 mg/L^6^. Cd(II) has been considered one of the most hazardous heavy metal ions to human health^7^. The high solubility of Cd(II) and its related compounds, compared with those of other heavy metals, means that it can effortlessly migrate to biological systems^8^. Cadmium ions have high toxicity, and carcinogenesis can occur even at low dosage^9^. In line with the World Health Organization (WHO), the maximum Cd(II) concentration in domestic water supplies should be 5 μg/L^10^. Mercury typically enters the environment via activities such as pharmaceutical processing, electroplating, mining and industrial effluent and further accumulates in organs and bodies, causing harm, especially to nerves and related systems^11^. Due to its high environmental mobility and the fact that Hg(II) has a unique position in the biogeochemical cycle, it accumulated inside organisms, and its impact magnifies when it moves up the food chain and ecosystem^12,13^. Accordingly, WHO has banned the presence of mercury in potable water and imposed a limit of 0.1 µg/L in effluents^14^. In conclusion, waters contaminated with zinc, cadmium and mercury ions can cause various adverse effects on living organisms; therefore, it is critical to remove them from wastewater before it is released into the environment^15^. Hence, the extraction of the zinc subgroup ions Zn(II), Cd(II) and Hg(II) from effluents is significant.

In contemporary times, various physical, chemical, biological, and electrochemical treatment technologies are employed to extract heavy metal ions from aqueous media, including adsorption, oxide reduction, ion exchange, precipitation, filtration, etc.^16^. These approaches, however, pose problems such as high expenses, the creation of harmful byproducts causing further pollution, and an inability to effectively remove ions if their concentrations are low^17^. Among these methods, adsorption has arisen and predominated, as it is simple to design, economical and highly effective^18^. The present work aims to describe, to the best of our knowledge, the novel application of a TLSB nanocomposite to remove zinc, cadmium and mercury ions in effluents.

Lignocellulose (LCS), a kind of polymer, is nontoxic, biocompatible, biodegradable, and environmentally friendly^19^. There are multiple oxygen moieties (–OH, –COO, –C=O, C–O–C, etc.) in its molecular chains^20^. However, the adsorption capacity of unmodified LCS is relatively low, and LCS possesses low selectivity for adsorbates. Thus, diverse functionalized LCS materials have been employed to extract heavy metal ions. Thiol group (–SH) sites typically bind strongly with heavy metals due to their chelation and coordination behavior. Thiol-lignocellulose (TL) derivatives possess numerous distinct advantages, such as controllable biodegradation, good adhesion, and targeting of heavy metals^21^. The introduction of thiol-containing groups to LCS increases the stability of complexed heavy metals, thus enhancing the sorption capacity as well as selectivity^22^. Sodium bentonite (SB) is the most widely used clay mineral and is composed of a sandwich structure that consists of two silica tetrahedron sheets sandwiching an aluminum octahedral sheet. SB has a good adsorption capacity because of its large surface area, large capacity for cation exchange and microlayered structure^23^. Recently, biodegradable natural polymer/clay nanocomposites have been investigated^24^. However, the adsorption and desorption by thiol-lignocellulose/sodium bentonite of heavy metal ions, particularly zinc, cadmium and mercury ions, have been rarely reported.

Polymer/clay nanocomposites are attracting considerable interest in the adsorption of heavy metals because they frequently exhibit a remarkably improved adsorption capacity and material properties. To the best of our knowledge, there are few studies focusing on the adsorption capacity of TLSB. The originality of this work is that it can be used to analyze the different adsorption capacities of a novel TLSB nanocomposite for the zinc subgroup ions Zn(II), Cd(II) and Hg(II); these ions are all located in the same group (IIB) and the 4th, 5th, and 6th periods, respectively, of the periodic table but have different ionic radii, electronegativities, valence-electron arrangements, soft and hard acid–base alkalinities, solubility product constants (*K*_sp_) of their hydroxides and sulfides, etc. Therefore, the effects of TLSB nanocomposite dosage, zinc subgroup ion concentration, solution pH, temperature, time of adsorption, different types and concentrations of desorption reagents, desorption temperature, and ultrasonic desorption time on the adsorption and desorption capacities of TLSB for zinc, cadmium and mercury ions were investigated. Furthermore, the adsorption kinetics, isotherms and thermodynamics of the TLSB nanocomposite were examined, and the mechanism of Zn(II), Cd(II) and Hg(II) adsorption was discussed as well. TLSB has a higher adsorption capacity than other reported materials. The obtained results offer a reference for the selection of TLSB nanocomposites as the appropriate material for Zn(II), Cd(II) and Hg(II) extraction; the regeneration ability of the nanocomposite was examined through several adsorption/desorption cycles, and the material exhibited good recycling performance.

## Results and discussion

### Characterization of thiol-lignocellulose/sodium bentonite (TLSB)

**Figure 1:**
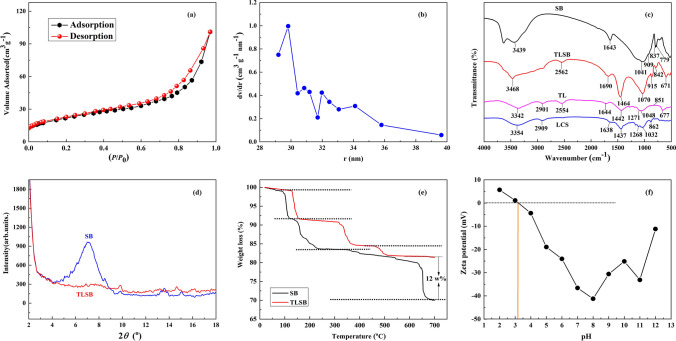
(**a**) Adsorption/desorption curves of N_2_ on TLSB. (**b**) Pore size distribution curve of TLSB. (**c**) FTIR spectra of SB, LCS, TL and TLSB. (**d**) XRD patterns of SB and TLSB. (**e**) TGA analyses of SB and TLSB. (**f**) Zeta potential measurements of TLSB at different pH values.

The physicochemical properties of the TLSB nanocomposite were observed with different characterization techniques. The content of thiol groups was 4.102% in the TLSB nanocomposite, as determined by the indirect iodometric method. No thiol groups were detected in the LCS or SB, indicating that the thiol groups were introduced into lignocellulose by modification. Qualitative information regarding the pore size distribution, pore volume and specific surface area of TLSB was provided by N_2_ adsorption/desorption isotherms at − 77 °C obtained by an ASAP 2020 Micromeritics surface area analyzer. The microscopic parameters of the specific surface area of TLSB were determined by the BET and Langmuir methods, whereas the pore volume and size distribution of TLSB were found by the BJH method^25^. The N_2_ adsorption/desorption isotherms and distribution of the pore size are summarized in Fig. 1a,b and Table 1, respectively. From Fig. 1a, it can be observed that TLSB exhibits a type (IV)-shaped isotherm; in the middle of the curve, there is an H4 hysteresis loop, demonstrating that the adsorbent is a typical mesoporous material. According to Fig. 1b and Table 1, the pore size distribution of TLSB is wide but nonuniform, with most of the pore sizes concentrated at approximately 32 nm. As shown in Table 1, the specific surface area of TLSB was measured using the Langmuir method (874.3 m^2^/g) and BET method (622.93 m^2^/g), and the average pore size (29.03 nm) and pore volume (5.809 cm^3^/g) were determined. The experimental results indicate that the specific surface area, pore volume and mean pore size of TLSB were enhanced to varying degrees after modification with thiol groups. The following adsorption experiment illustrates that TLSB has a large capacity to adsorb heavy metal ions due to these microscopic parameters being enhanced.

**Table 1:** Surface area and pore structure parameters of SB, LCS/SB, and TLSB.

Sample	*S*_BET_ (m^2^/g)	*S*_Langmuir_ (m^2^/g)	*V*_Pore_ (cm^3^/g)	*V*_Mic_ (cm^3^/g)	*R*_Mic_ (nm)	*V*_Mes_ (cm^3^/g)	*R*_Mes_ (nm)	*R*_Ave_ (nm)
SB	61.4	137.52	1.058	0.024	–	0.030	–	204.05
LCS/SB	213.50	378.06	3.664	1.051	1.98	2.217	45.93	31.82
TLSB	622.93	874.30	5.809	0.771	1.202	4.036	42.77	29.03

FTIR (Fig. 1c) was utilized to examine the characteristic chemical structures of SB, TLSB, TL and LCS within the wavenumber range of 500–4000 cm^−1^. As shown in Fig. 1c, several absorbance peaks can be found in the FTIR spectrum of LCS, such as the –OH bending vibration of water (3354 cm^−1^); the C–H stretching vibration of methyl and methylene (2909 cm^−1^); the O–C=O stretching vibration of –COOH (1638 cm^−1^); the C–O–C symmetric stretching vibration (1437 cm^−1^); the C–O symmetric stretching vibration (1268 cm^−1^); the C–O out-of-plane bending vibration (1032 cm^−1^); and the C–H vibration of benzene and aromatic rings (862 cm^−1^)^26^. These absorption peaks were amplified after thiol group modification and shifted to wavenumbers of 3342 cm^−1^, 2901 cm^−1^, 1644 cm^−1^, 1442 cm^−1^, 1271 cm^−1^ and 1048 cm^−1^, respectively. The particularly strong peaks at 2554 cm^−1^ and 667 cm^−1^ are caused by the –SH stretching vibration and C–S bending vibration in the FTIR spectrum of TL^27^. All of these results proved that thiol groups were incorporated into the LCS chemical structure and that TL was prepared successfully.

Referring to the FTIR spectrum of SB, the –OH bending vibration of H_2_O in the SB interlayer at 3439 cm^−1^ shifted to a higher wavenumber of 3468 cm^−1^ after intercalation. The characteristic adsorption peaks at 1643 cm^−1^ of SB and 1644 cm^−1^ of TL moved to 1690 cm^−1^ after intercalation, which proved that the C–O stretching vibration absorption peak of TL covered the –OH characteristic absorption peak of H_2_O in the SB interlayer completely. The –COO characteristic absorption peak at 1442 cm^−1^ and the –OH absorption peak corresponding to carboxyl groups shifted to 1464 cm^−1^ and 1070 cm^−1^, respectively^28^. All these peak shifts occurred after TLSB was prepared. The telescopic vibration adsorption peaks at 909 cm^−1^, 837 cm^−1^ and 779 cm^−1^ correspond to Al–O–H, Si–O and Mg–O–H in the SB chemical structure, respectively, and they were clearly abated, as evident in the spectra of TLSB. In the FTIR spectrum of TL, the S–H characteristic adsorption vibration peak at 2554 cm^−1^ and the C–S stretching vibration peak at 677 cm^−1^ increased to 2562 cm^−1^ and decreased to 671 cm^−1^, respectively, in the TLSB FTIR spectrum^29^. As shown in the FTIR spectrum, TL entered into the interlayer space of SB, and the activated TLSB adsorption sites were present not solely on –COO– but also on many other sites, such as Al–O, Si–O, C–O–C, C=O, –OH, –SH, etc., which enhanced the adsorption capacity for heavy metals by the TLSB nanocomposite.

XRD analysis of TLSB and SB was performed with an X-ray power diffractometer (XRD) with Kα radiation in the 2*θ* range 2°–15° (X’pero PRO, Almelo, The Netherlands) at a voltage of 40 kV and current of 30 mA, and the results are presented in Fig. 1d. The typical (Bragg) diffraction peak of SB is 6.94°, corresponding to a basal spacing of 1.27 nm, which demonstrates that SB has a typical nanostructure. This characteristic peak greatly diminished and almost disappeared after TLSB was intercalated into SB, and the interlayer spacing of TLSB shifted to 1.94 nm. The results illustrate that the SB interlayers were dispersed into TL due to the expansion and exfoliation of SB in the stacking direction and that the SB nanostructure was destroyed when the TL molecules were intercalated into the TLSB layers^30^. It is evident that a new type of intercalated-exfoliated nanostructure may have been created in the TLSB nanocomposite by the intercalation of TL into SB, increasing the active adsorption sites and high adsorption capacity for heavy metal ions of the nanocomposite^31^.

Thermogravimetric analysis (TGA) data of the samples were recorded at a heating rate of 10 °C per min in the temperature range of 20–700 °C on a Perkin Elmer Instrument. The thermal decomposition of SB and TLSB is shown in Fig. 1e. The mass loss at the highest temperature corresponds to the degradation of the main chains; thus, this temperature reflects the thermal stability of the polymer skeleton. The thermal decomposition of SB is observed to occur in three stages in Fig. 1e. The first stage is attributed to the loss of surface adsorbed water (approximately 8%) at a temperature of approximately 110 °C. In the temperature range of 150–230 °C, a weight loss of approximately 9% is apparent due to the removal of adsorbed water between interlayers. The maximum decomposition rate occurred at approximately 660 °C with a total weight loss of 16%, attributed to the loss of interlamellar hydroxyl groups. TLSB also exhibited three distinct stages. The first stage occurred at approximately 155 °C and was due to the evaporation of adsorbed water associated with the nanocomposite, with a TLSB weight loss rate of 6%. The second weight loss stage took place at approximately 340 °C because a small amount of organic matter decomposed. The final weight loss of approximately 4% began at 500 °C due to the grafted chemical bonds being broken down and the thermal degradation of polymers^32^. From these data, it is apparent that SB contributed approximately 12% to the total mass of TLSB, and TLSB showed a lower rate of weight loss within the temperature range of 20–700 °C, implying greater thermal robustness.

To determine the point of zero charge (PZC) of TLSB, the surface charge was determined by a zeta potential analyzer (NanoPlus Series-2, USA) at room temperature. The measurement of PZC was conducted by adjusting the pH of a 50 mL sample with 0.01 M HCl or 0.01 M NaOH solution to a value between 2 and 12. TLSB was added, and the final pH was tested after 48 h under agitation. The results of the surface charge analysis of TLSB as a function of pH are displayed in Fig. 1f. The negative surface charge was found to increase steadily with increasing pH until pH 8, at which point the highest negative charge (− 41.22 mV) was recorded. Beyond pH 8, the surface charge on TLSB was unstable. Additionally, the pH value of 3.15 can be considered its PZC. When the pH was higher than the PZC, the net surface charge was negative, which was beneficial for cation adsorption^33^. In the pH range below 3.15, the net surface charge would be positive, which was not favorable for metal ion adsorption. The PZC value for TLSB indicated that the adsorption of zinc subgroup ions will occur at pH ≥ 3.15.

**Figure 2:**
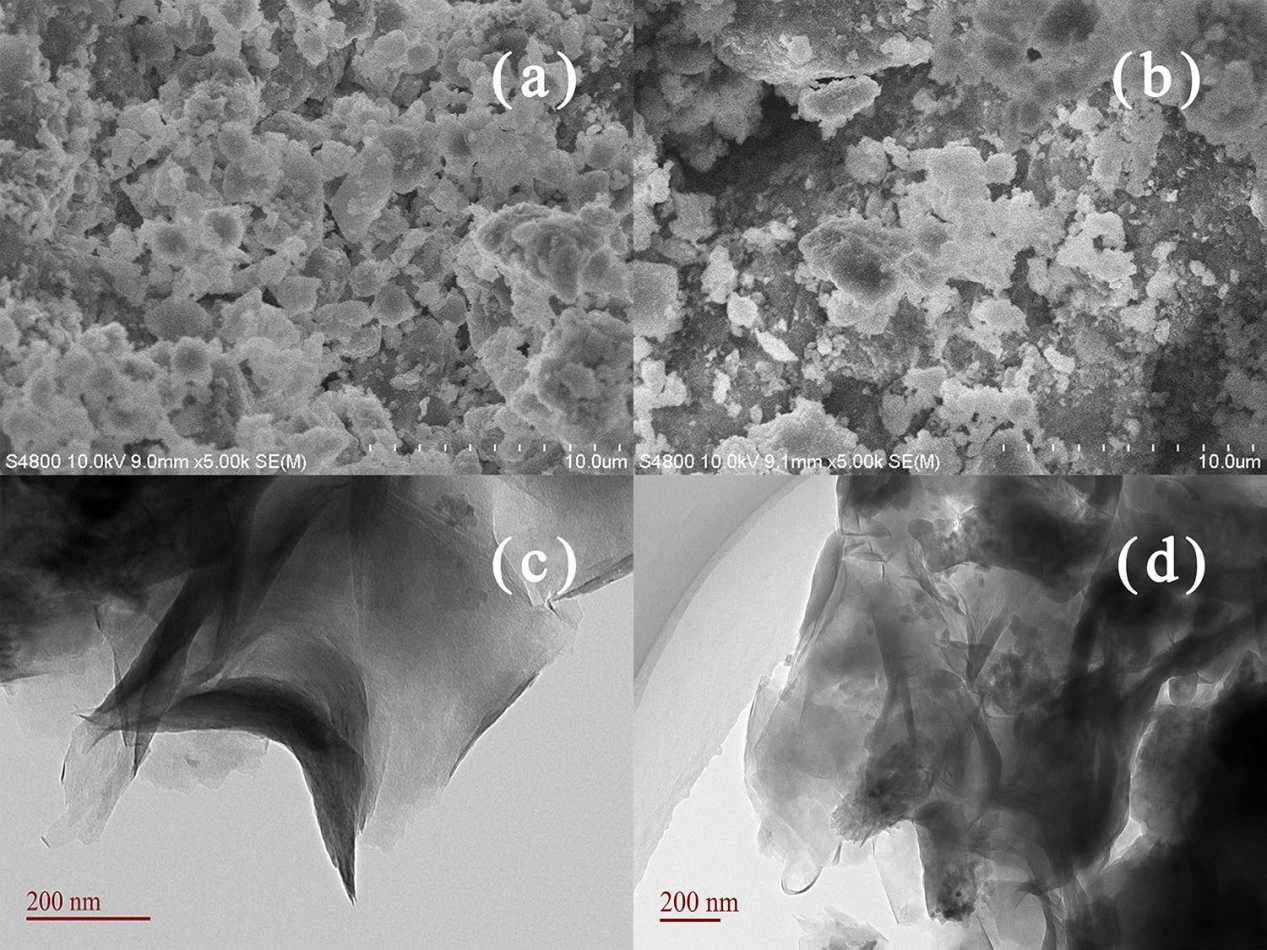
SEM images of SB (**a**) and TLSB (**b**) nanocomposites. TEM images of SB (**c**) and TLSB (**d**) nanocomposites.

SEM analysis of SB and TLSB were conducted with a HITACHI S-4800 microscope (Tokyo, Japan) and S-3400/NOXFORD-XMasN (HORIBA, Japan). As presented in the SEM image of SB (Fig. 2a), we can see that the pristine SB particles display comparative density, sheet-stacked layers and a stretched surface in their structure. However, the introduction of TL molecules destroyed the crystalline structure of SB, and TLSB (Fig. 2b) appeared to have a well-developed coarse porous surface with irregular clusters of dispersed stacked sheets. The incorporation of TL produced numerous cavities and a comparatively loose surface, which eventually led to an increase in the contact area and activated sites for the adsorption of heavy metals from effluents^34^. Table 1 further supports the explanation above. Therefore, these phenomena indicated that almost all of the TL was intercalated into the SB interlayers, forming the intercalated-exfoliated TLSB nanocomposite, which is in accordance with the results of the XRD patterns^35^.

Meanwhile, we used a TEM instrument (JEM-2010, Tokyo, Japan) at 75–100 kV to determine the morphologies and structures of TLSB and SB. Combining XRD and TEM is a powerful method for characterizing the structure of polymer/clay composites. The comparison of the TEM images of SB and TLSB (Fig. 2c,d) reveals that the dark lines in the image are the intersections of the clay sheets, and the spaces between the dark lines are TL. Almost all the TL was embedded within SB interlayers with the destruction of the crystalline structure of SB, and the polymer matrix was well dispersed in the clay interlayers. These phenomena suggested that TL was introduced into SB and formed a disordered intercalated structure^36^.

## Adsorption studies

### Effect of heavy metal concentration

**Figure 3:**
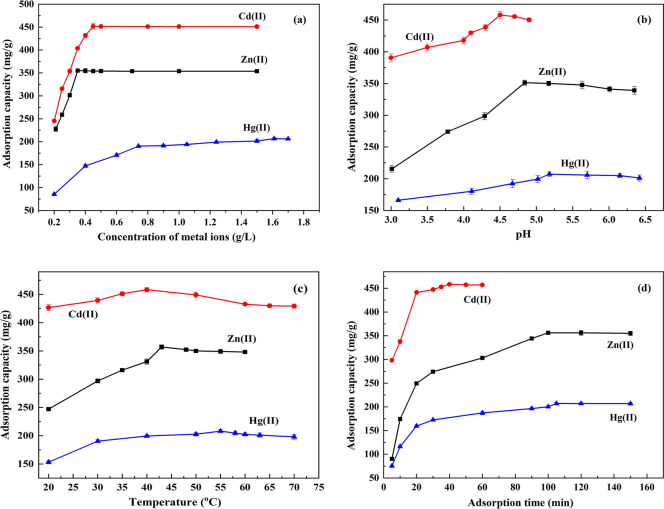
(**a**) Effect of heavy metal concentration on the adsorption capacity of TLSB. (**b**) Effect of solution pH on the adsorption capacity of TLSB. (**c**) Effect of adsorption temperature on the adsorption capacity of TLSB. (**d**) Effect of adsorption time on the adsorption capacity of TLSB.

The impact of the concentrations of the inspected metals on the equilibrium behavior of TLSB as an adsorbing material for Zn(II), Cd(II) and Hg(II) is clarified in Fig. 3a (Zn(II) adsorption experiments: concentration range 0.20–1.50 g/L, sample dosage 0.05 g, pH 4.84, adsorption temperature 43 °C, adsorption time 100 min; Cd(II) adsorption experiments: concentration range 0.20–1.50 g/L, sample dosage 0.05 g, pH 4.52, adsorption temperature 40 °C, adsorption time 40 min; Hg(II) adsorption experiments: concentration range 0.20–1.70 g/L, sample dosage 0.05 g, pH 5.18, adsorption temperature 55 °C, adsorption time 105 min). It is noted that the adsorption capacity of the zinc subgroup ions Zn(II), Cd(II) and Hg(II) on TLSB increased in tandem with metal concentration until it reached the maximum capacity. The maximal adsorption capacities of TLSB are 357.29 mg/g, 458.32 mg/g and 208.12 mg/g for Zn(II), Cd(II) and Hg(II), respectively, at the optimum metal concentrations of 0.34 g/L for Zn(II), 0.45 g/L for Cd(II) and 1.61 g/L for Hg(II). This result may be attributed to the fact that when the concentration was lower at the beginning, the adsorption of heavy metals occurred on the exterior surface of the TLSB, yet with rising concentration, the heavy metal ions were able to overcome the resistance due to mass transfer from the aqueous phase to the solid phase and subsequently penetrated into the inside structure, occupying increasingly numerous adsorption sites on TLSB^37,38^. The adsorption capacity did not change significantly after an additional increase in metal concentration because there were no additional adsorption sites for heavy metals on the surface of TLSB^39^.

Based on the hard and soft acids and bases (HSAB) theory, hard acids have a preference for hard bases, and vice versa^40^. Hard acids are predominantly adsorbed by small ligands that contain oxygen and moieties such as hydroxyl and carboxyl groups interacting electrostatically. For soft acids, adsorption principally occurs with thiol-containing ligands. The thiol groups (soft bases) in TLSB offered strong bonding sites for soft metals such as Cd(II) and Hg(II) ions while providing weak bonding sites for hard acids such as Zn(II)^41^. As demonstrated in Fig. 3a, the adsorption capacity of TLSB for Cd(II) is more significant than that for Zn(II). It is noted that the adsorption capacity for Hg(II) was not as good as expected, and this phenomenon can be understood from the following observations: (1) The ionic radii of the ions increase in the order Zn(II) (74 pm) < Cd(II) (95 pm) < Hg(II) (102 pm), while the average pore size of the TLSB nanocomposite is 29.03 nm. The ion size of Hg(II) is larger than those of Zn(II) and Cd(II); Zn(II) and Cd(II) can easily enter the interior of TLSB to bind with active sites, while entry is difficult for Hg(II), thus reducing the adsorption capacity^42^. (2) The observed adsorption capacity may be related to the ion-exchange mechanism. The ion-exchange capacity is related to the radius of hydrated cations. The radius of Hg(II) is so large that it could not be easily replaced by the ions in TLSB. (3) Deposition after the adsorption of Hg(II) by TLSB probably blocked some of the surface pores, resulting in a decrease in pore volume and surface area that may have reduced the adsorption capacity for Hg(II) of TLSB^43^. In summary, Zn(II), Cd(II) and Hg(II) reached adsorption equilibrium at the optimum concentrations of 0.34 g/L, 0.45 g/L and 1.61 g/L, respectively.

### Effect of pH

The surface charge of the adsorbent and the hydrolyzed form of metal ions were related to the solution pH, thus it is clearly that the solution pH was strongly affect the removal of heavy metal ions from aqueous. The PZC value was the net surface charge of a material in aqueous solution which will along with pH to explain the removal capacity of heavy metal ions by adsorbent. The PZC value of TLSB was 3.15 in this work (Fig. 1f).Therefore, the removal of metal ions was favored at pH > PZC, where the surface of TLSB is negatively charged, owing to the deprotonation of carboxylic acid groups^44^. Figure 3b shows that the impact of pH on TLSB was examined in the ranges of 3.01–6.35, 3.00–4.91, and 3.10–6.42 for Zn(II), Cd(II) and Hg(II), respectively (Zn(II) adsorption experiments: sample dosage 0.05 g, concentration 0.34 g/L, adsorption temperature 43 °C, adsorption time 100 min; Cd(II) adsorption experiments: sample dosage 0.05 g, concentration 0.45 g/L, adsorption temperature 40 °C, adsorption time 40 min; Hg(II) adsorption experiments: sample dosage 0.05 g, concentration 1.61 g/L, adsorption temperature 55 °C, adsorption time 105 min). As noted from Fig. 3b, the pH value does indeed markedly impact the adsorption process of TLSB for Zn(II), Cd(II) and Hg(II). The capacity for adsorption rose from 215.30 to 351.29 mg/g for Zn(II), 390.51 to 457.91 mg/g for Cd(II), and 166.08 to 207.22 mg/g for Hg(II) as the pH rose. At higher pH values, the adsorption of ions decreased to different degrees.

Figure 3b shows that the adsorption capacities of Zn(II), Cd(II) and Hg(II) were enhanced with increased pH and then decreased slightly or remained unchanged as the solution pH rose. Heavy metal adsorption depends on the interactions among the heavy metal ions and the moieties on the exterior of TLSB, which is impacted by the solution pH value^45^. At low pH, the competition between H^+^ ions and adsorption sites on the TLSB surface became more intense as the H^+^ concentration rose; thus, the adsorption capacity decreased^46^. In the pH range of 3.01–4.84, Zn(II) exists as a dissolved metal. For pH values above 4.84, Zn(II) may precipitate out as ZnOH and Zn(OH)_2_ (*K*_sp_ 1.2 × 10^–17^), decreasing the percentage of adsorption^47^. The hydrated radius of Cd(II) (0.426 nm) is smaller than that of Zn(II) (0.43 nm), and the smaller an ion's hydration radius is, the tighter and more powerful surface adsorption will be. Furthermore, Cd(II) has a higher electronegativity than Zn(II). Therefore, the adsorption capacity for Cd(II) is greater than that for Zn(II). An increase in solution pH above 4.52 leads to hydrolysis, forming precipitation in the form of cadmium hydroxide Cd(OH)_2_ (*K*_sp_ 5.27 × 10^–15^) in the metal solution. Therefore, the adsorption efficiency of TLSB for Cd(II) decreased as the pH increased^48^.

For Hg ions, low-pH conditions resulted in high competition between Hg ions and H^+^ ions that resulted from the protonation of TLSB and led to lower adsorption under lower pH^49^. At a pH above 5.18, the hydrolysis of Hg ions to [Hg_2_OH]^+^ and Hg(OH)_2_ (*K*_sp_ 3.0 × 10^–26^) caused a decrease in the adsorption capacity of Hg by TLSB^50^. However, the solution pH was not significant for Hg(II) due to the high affinity of Hg(II) toward thiol groups on TLSB. The phenomenon may be explained by taking the solubility products (*K*_*sp*_) of HgS (4.0 × 10^–53^), CdS (3.60 × 10^–29^) and ZnS (1.2 × 10^–23^) into account. Adsorption at pH values greater than 5.18 was not evaluated for Hg(II) due to the formation of hydrolyzed metal species [M(OH)_2_]. Therefore, pH 4.84, 4.52 and 5.18 were selected as the optimum pH values in the following studies.

### Effect of adsorption temperature

The adsorption characteristics of TLSB at different temperatures are shown in Fig. 3c (Zn(II) adsorption experiments: sample dosage 0.05 g, concentration 0.34 g/L, pH 4.84, adsorption time 100 min; Cd(II) adsorption experiments: sample dosage 0.05 g, concentration 0.45 g/L, pH 4.52, adsorption time 40 min; Hg(II) adsorption experiments: sample dosage 0.05 g, concentration 1.61 g/L, pH 5.18, adsorption time 105 min). Figure 3c shows that the adsorption capacities of TLSB increased gradually and reached peak values of 247.00 mg/g and 357.29 mg/g for Zn(II) at temperatures of 20 °C and 43 °C, 426.68 mg/g and 458.30 mg/g for Cd(II) at temperatures of 20 °C and 40 °C, and 153.2 mg/g and 208.12 mg/g for Hg(II) at temperatures of 20 °C and 55 °C, which demonstrates that elevated temperature is more favorable for the adsorption process, indicates that heat-absorbing properties are intrinsic to the adsorption mechanism, and further affirms that this reaction is endothermic^51^. With rising temperature, the diffusion rate of heavy metal ions crossing over the outside boundary layer and interior pores of adsorbent particles rises and will not only speed up the diffusion rate from the solution to the surface of TLSB but also accelerate the complexation of heavy metals with TLSB functional groups^52^. On the one hand, a slight reduction in adsorption capacity at elevated temperatures may occur because the chemical adsorption process is exothermic, spontaneous and entropy reducing. On the other hand, the adsorption capacity decreased due to the organic matter of TLSB decomposing and breaking down at high temperature^53^ (Fig. 1e).

### Effect of adsorption time

It is important to determine the effect of the amount of time required to reach adsorption equilibrium when conducting a batch of experiments. The effect of adsorption time on removal is shown in Fig. 3d (Zn(II) adsorption experiments: sample dosage 0.05 g, concentration 0.34 g/L, pH 4.84, adsorption temperature 43 °C; Cd(II) adsorption experiments: sample dosage 0.05 g, concentration 0.45 g/L, pH 4.52, adsorption temperature 40 °C; Hg(II) adsorption experiments: sample dosage 0.05 g, concentration 1.61 g/L, pH 5.18, adsorption temperature 55 °C). The results show that Zn(II), Cd(II) and Hg(II) removal follows a gently increasing trend with time and that Zn(II), Cd(II) and Hg(II) adsorption on TLSB is rapid in the early process of adsorption. As presented in Fig. 3d, the adsorption capacities of the ions increased rapidly from 90.13 to 356.13 mg/g for Zn(II), from 298.06 to 458.41 mg/g for Cd(II), and from 75.29 to 206.95 mg/g for Hg(II) as the adsorption time increased. Early in the process of adsorption, the high solution concentration set up a steep concentration gradient, facilitating diffusion to the TLSB nanostructure, which resulted in rapidly increasing adsorption capacity^54^. After that, the adsorption sites were occupied completely, and there were no more adsorption sites for more heavy metals, so the adsorption process slowed^55^. Hence, to maintain adsorption equilibrium, durations of 100 min, 40 min and 105 min were considered sufficient to achieve the desired efficiency.

### Adsorption kinetics

Predictions of adsorption rates provide important information regarding adsorption mechanisms. To evaluate the adsorption mechanism onto TLSB, the experimental data at various adsorption times corresponding to the changes in adsorption capacity were fitted using the pseudo-first-order model, pseudo-second-order model, intraparticle diffusion model, double constant equation model and Elovich model, all of which are described in equations (Eq. 1–5)^56–60^. Kinetic experiments were carried out by the following optimal operating conditions: (1) an equilibrium concentration of 0.34 g/L and a pH of 4.84 for Zn(II) at 43 °C; (2 ) an equilibrium concentration of 0.45 g/L and pH 4.52 for Cd(II) at 40 °C; and (3) an equilibrium concentration of 1.61 g/L and pH 5.18 for Hg(II) at 55 °C.

1 $$ \lg (q_{e} - q_{t,1} ) = \lg q_{e} - \frac{{k_{1} t}}{2.303} $$

2 $$ \frac{t}{{q_{t,1} }} = \frac{1}{{k_{2} q_{e}^{2} }} + \frac{t}{{q_{e} }} $$

$$ q_{t} = k_{i} t^{0.5} $$

$$ \ln q_{t} = \ln A + B\ln t $$

5 $$ q_{t} = \frac{1}{\beta }\ln (\alpha \beta ) + \frac{1}{\beta }\ln t $$

where *q*_e_ and *q*_t_ are the amounts of heavy metal ions adsorbed (mg/g) at equilibrium and at time *t* (min), respectively; *k*_1_ (min^−1^) is the pseudo-first-order rate constant; *k*_2_ [g·(mg/min)^−1^] is the rate constant of the pseudo-second-order adsorption kinetic equation; *k*_*i*_ [mg/(g min^1/2^)] is the intraparticle diffusion rate constant; *A* and* B* are the double constant equation model constants; *α* [mg/(g min)] is the initial adsorption rate; and *β* (g/mg) is related to the surface coverage and activation energy for chemisorption.

**Figure 4:**
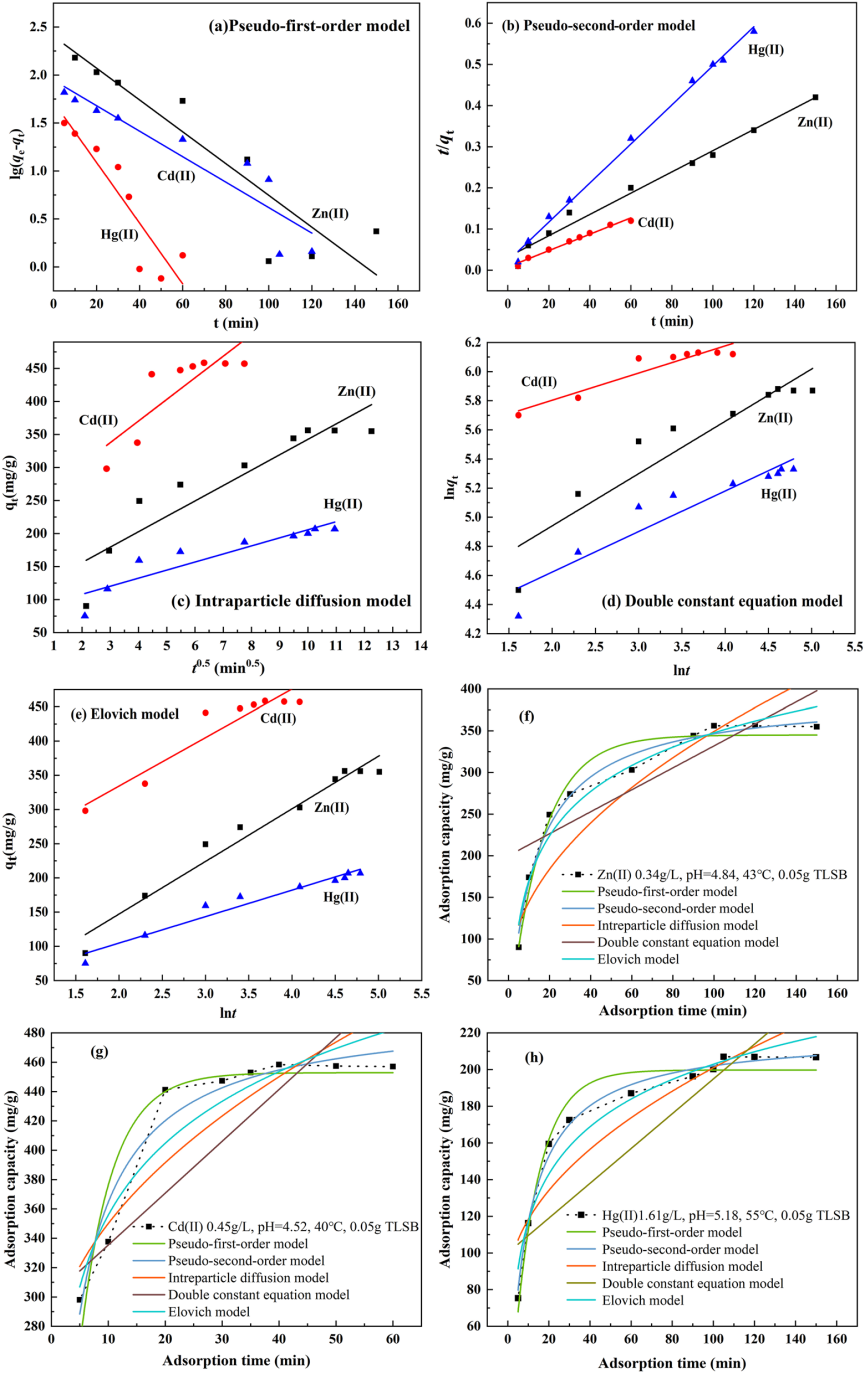
Linear fitting curves of adsorption kinetics equations to the experimental data. (**a**) pseudo-first-order model, (**b**) pseudo-second-order model, (**c**) intraparticle diffusion model, (**d**) double constant equation model, (**e**) Elovich model. Adsorption kinetics-related nonlinear fitting curves. (**f**) Zn(II) at pH 4.84, 0.34 g/L, 43 °C, 0.05 g TLSB, (**g**) Cd(II) at pH 4.52, 0.45 g/L, 40 °C, 0.05 g TLSB, and (**h**) Hg(II) at pH 5.18, 1.61 g/L, 55 °C, 0.05 g TLSB.

The Zn(II), Cd(II) and Hg(II) experimental adsorption rate data were fitted to the five kinetic models. All kinetics data for adsorption onto TLSB calculated from the related linear and nonlinear fitting curves are shown in Fig. 4 and Table 2. The correlation coefficient *R*^2^ was used to compare the fitting quality of the kinetic curves. Figure 4a shows a plot of lg(*q*_e  _− *q*_t_) as a function of time according to the linear pseudo-first-order model. The low values of *R*^2^ (0.4525 for Zn(II), 0.4932 for Cd(II) and 0.8298 for Hg(II) in Table 2) show that the adsorption of the three zinc subgroup ions did not follow the pseudo-first-order model. Similarly, Fig. 4b presents the plot of (*t*/*q*_t_) versus *t* in the linear pseudo-second-order model. The magnitudes of the adsorption capacities (357.29 mg/g for Zn(II), 458.32 mg/g for Cd(II), 208.12 mg/g for Hg(II)) according to this model were found to be very close to the experimental *q*_max–cal_ values of 366.83 mg/g for Zn(II), 471.23 mg/g for Cd(II), and 205.11 mg/g for Hg(II).and the *R*^2^ values were 0.9947, 0.9926, and 0.9956 for Zn(II), Cd(II) and Hg(II), respectively (Table 2), all close to 1.0, which suggests that the pseudo-second-order model fit the adsorption process well^61^. In Fig. 4c, the fit of the intraparticle diffusion model was determined by the plot of *q*_t_ versus *t*^0.5^. The *R*^2^ values were 0.8431 for Zn(II), 0.9186 for Cd(II), and 0.9218 for Hg(II) (Table 2), implying that the adsorption did not follow this model. The double constant equation model is also called the Freundlich modified formula, which is a more complex kinetic empirical formula of the reaction process. From Fig. 4d and Table 2, it can be seen that the *R*^2^ values were also lower than those of the pseudo-second-order model. In Fig. 4e, *q*_t_ was plotted versus ln*t* according to the Elovich kinetics model. *R*^2^ was 0.9022, 0.9496, and 0.9773 for Zn(II), Cd(II) and Hg(II) ions (Table 2), indicating that the adsorption sites were heterogeneous and that a variety of activation energies occurred during the adsorption process; that is, the adsorption process was also partially modeled by the Elovich model. A comparison of the *R*^2^ values vividly shows that the adsorption kinetics followed the pseudo-second-order model, as this model exhibited the highest *R*^2^, which is consistent with the results obtained in the adsorption kinetics-related nonlinear fitting curves of Zn(II), Cd(II), and Hg(II), respectively, in Fig. 4f, h. Overall, it is suggested that the kinetics of zinc subgroup ions, which follow the pseudo-second-order model, are controlled by a chemisorption process. In this case, it can be observed that the sorption of Zn(II), Cd(II) and Hg(II) ions involved precipitation and chemical adsorption interactions, such as inner-sphere ion exchange, complexation, chelation and electrostatic interactions.

**Table 2:** *R*^2^ and constant values for the different adsorption kinetics models of Zn(II), Cd(II) and Hg(II) on TLSB.

Metals	Parameters	Pseudo-first-order model		Pseudo-second-order model		Intraparticle diffusion model		Double constant equation model		Elovich model	
Zn(II)	*R*^2^	0.4525		0.9947		0.8431		0.8772		0.9022	
	Constants	*k*_1_	0.0201 min^−1^	*k*_2_	0.308 g (min/min)^−1^	*k*_i_	0.339 mg/(g min^0.5^)	*A*	0.1980	*α*	9.17 mg/(g min)
		*q*_e_	247.08 mg/g	*q*_e_	366.83 mg/g			*B*	0.0963	*β*	0.0211 g/mg
Cd(II)	*R*^2^	0.4932		0.9926		0.9186			0.8589	0.9496	
	Constants	*k*_1_	0.011 min^−1^	*k*_2_	0.587 g (min/min)^−1^	*k*_i_	0.116 mg/(g min^0.5^)	*A*	0.0880	*α*	8.01 mg/(g min)
		*q*_e_	310.41 mg/g	*q*_e_	471.23 mg/g			*B*	0.1007	*β*	0.008 g/mg
Hg(II)	*R*^2^	0.8298		0.9956		0.9218			0.8779	0.9773	
	Constants	*k*_1_	0.014 min^−1^	*k*_2_	0.291 g (min/min)^−1^	*k*_i_	0.241 mg/(g min^0.5^)	*A*	0.0097	*α*	18.25 mg/(g min)
		*q*_e_	274.10 mg/g	*q*_e_	205.11 mg/g			*B*	0.6811	*β*	0.0019 g/mg

### Adsorption isotherms

Adsorption isotherms describe how different kinds of pollutants interact with adsorbents and are thus very important in the clarification of adsorption mechanisms as well as in determining the equilibrium adsorption capacity and its influence on the surface properties of adsorption. Hence, adsorption isotherms are essential in the design of batch adsorption systems. The adsorption of the zinc subgroup ions Zn(II), Cd(II) and Hg(II) on TLSB was investigated in monocomponent aqueous solutions. The Langmuir, Freundlich, Temkin, and Dubinin–Radushkevich (D–R) models were selected to fit the equilibrium data obtained from the batch adsorption experiments by varying the concentrations of zinc subgroup ions from 0.20 to 1.71 g/L. The Langmuir equation (Eq. 6) can be written as follows^61–64^:

6 $$ \frac{{C_{e} }}{{q_{e} }} = \frac{1}{{bq_{m} }} + \frac{{C_{e} }}{{q_{m} }} $$

The essential characteristics of the Langmuir isotherm can be expressed by a dimensionless constant called the equilibrium parameter *R*L, which is defined by the following equation (Eq. 7):

$$ R_{{\text{L}}} = \frac{1}{{1 + K_{{\text{L}}} C_{0} }} $$

The Freundlich (Eq. 8), Temkin (Eq. 9) and D–R isotherm equations (Eqs. 10, 11) can be represented as follows:

8 $$ \ln q_{e} = \ln k_{f} + \frac{1}{n}\ln C_{e} $$

$$ q_{e} = \frac{RT}{{b_{t} }}\ln \alpha_{t} + \frac{RT}{{b_{t} }}\ln C_{e} $$

$$ \ln q_{e} = \ln q_{\max } - B\varepsilon^{2} $$

$$ \varepsilon = RT\ln \left( {1 + \frac{1}{{C_{e} }}} \right) $$

where *q*_max_ (mg/g) is the monolayer saturation adsorption capacity; *C*_e_ (mg/L) is the concentration of metal ions at equilibrium; *K*_*L*_ (L/mg) is the Langmuir constant related to the adsorption capacity; the value of *R*_L_ indicates the nature of the isotherm as unfavorable (*R*_L_ > 1), linear (*R*_L_ = 1), favorable (0 < *R*_L_ < 1), or irreversible (*R*_L_ = 0); *K*_f_ is the Freundlich constant, 1/*n* is the value used to indicate the heterogeneity of the interface; *q*_e_ (mg/g) is the adsorption capacity at equilibrium; *R* is the ideal gas constant [8.314 J/(mol K)]; *T* (K) is the absolute temperature of the adsorption process; *α*_t_ (L/g) and *b*_t_ (J/mol) are Temkin isotherm constants; *B* is the D–R constant, and *ε* is the Polanyi potential, which can be calculated from Eq. (11).

**Figure 5:**
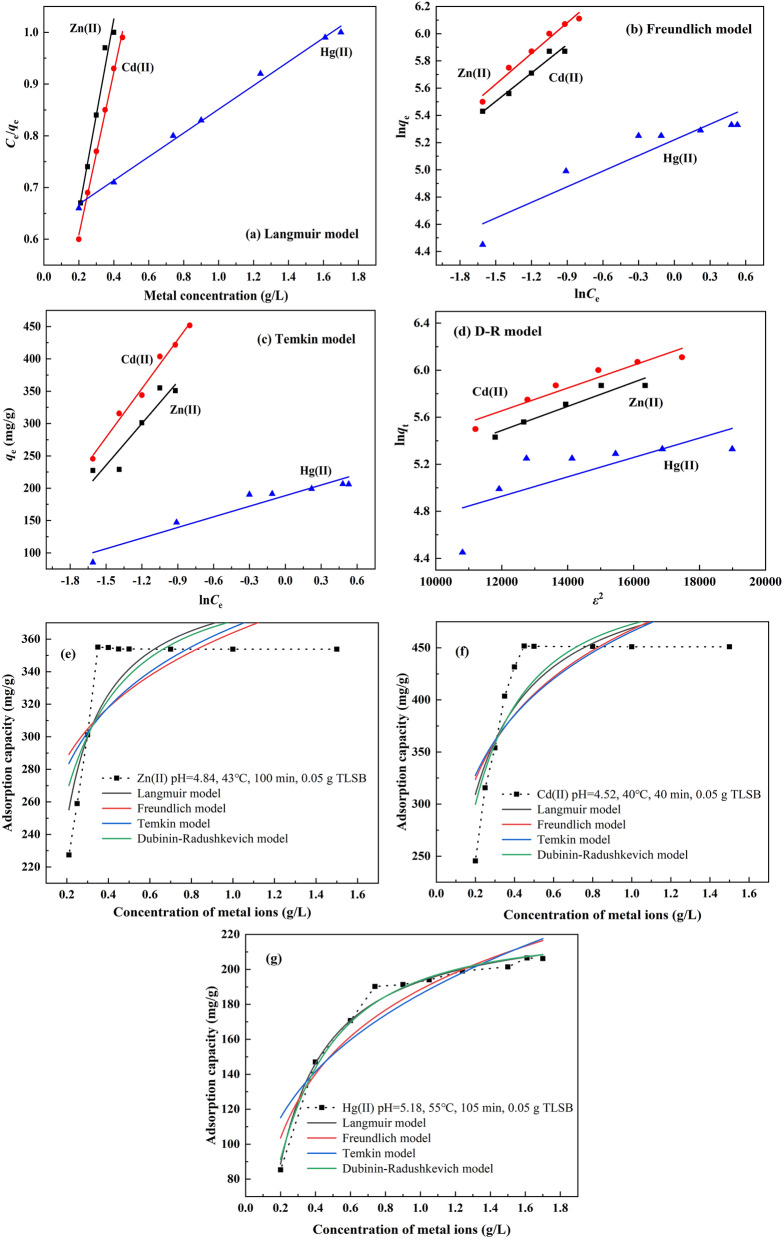
Linear fitting curves of adsorption isotherm models to the experimental data. (**a**) Langmuir, (**b**) Freundlich, (**c**) Temkin, and (**d**) D–R models. Nonlinear fitting curves for the adsorption isotherms of (**e**) Zn(II) at pH 4.84, 43 °C, 100 min, 0.05 g TLSB, (**f**) Cd(II) at pH 4.52, 40 °C, 40 min, 0.05 g TLSB, and (**g**) Hg(II) at pH 5.18, 55 °C, 105 min, 0.05 g TLSB.

Plots of the linear and nonlinear fitting curves of the Langmuir, Freundlich, Temkin and D–R isotherm models for the adsorption of Zn(II), Cd(II) and Hg(II) onto the surface of TLSB are presented in Fig. 5. The isotherm parameters are listed in Table 3. From Fig. 5a and Table 3, it is clear that the Langmuir plot had good linearity, since the correlation coefficient *R*^2^ value was 0.9956 for Zn(II), 0.9964 for Cd(II), and 0.9950 for Hg(II), which were closer to 1.0 than the values of the other models. The maximum monolayer adsorption capacity values calculated from the Langmuir model (*q*_max–cal_) were 351.36 mg/g, 465.29 mg/g, and 211.02 mg/g for Zn(II), Cd(II) and Hg(II), respectively, which were found to be very close to the experimental *q*_max_ values. Furthermore, the values of *R*_L_ for the adsorption of zinc subgroup ions on TLSB were all between 0 and 1, confirming that TLSB was favorable for adsorption under the employed adsorption conditions. The *R*^2^ of the Freundlich plot (Fig. 5b) was lower than that of the Langmuir plot, and the value of 1*/n* indicated the favorability of the adsorption of Zn(II), Cd(II) and Hg(II). The Temkin isotherm constant *b*_t_ was 51.02 J/mol for Zn(II), 35.66 J/mol for Cd(II), 39.07 J/mol for Hg(II), indicating the physicochemical nature of the adsorption process (Fig. 5c). Based on the *R*^2^ values (Table 3), the Temkin model did not perfectly fit the adsorption data. The apparent free energy of adsorption *ε* provides insight into the adsorption mechanism, which was calculated by the D–R isotherm model. It can be seen from Fig. 5d and Table 3 that the obtained mean free energy *ε* was 8.58 kJ/mol, 10.26 kJ/mol, and 9.67 kJ/mol for Zn(II), Cd(II) and Hg(II), respectively, which may be attributed to the fact that the adsorption process might be chemical adsorption. It is obviously that the Langmuir model describes the adsorption of zinc subgroup ions onto the TLSB nanocomposite much better than the other models, which means that the surface of TLSB have formed a monolayer coverage of Zn(II), Cd(II) and Hg(II), and homogenous in nature. This result also means that every adsorption site of TLSB has the same adsorption energy, and the *K*_L_ (the energy constant related to the heat of adsorption) values were calculated to be 0.2130 L/mg for Zn(II), 0.1744 L/mg for Cd(II), 0.1996 L/mg for Hg(II). Therefore, the adsorption of Zn(II), Cd(II) and Hg(II) through M–O and M–S linkages forms a layer between the metallic layers of TLSB that corresponds to the monolayer of the Langmuir isotherm model, which conforms to the results obtained in the adsorption isotherm nonlinear fitting curves of Zn(II), Cd(II), and Hg(II), respectively, in Fig. 5e, g. Table 4 lists a comparison of the maximum monolayer adsorption capacities of previously reported adsorbents. It can be concluded that the TLSB prepared in this work showed a higher adsorption capacity than those reported in the literature. Thus, it is noteworthy that TLSB is an excellent adsorbing material for the adsorption of the zinc subgroup ions Zn(II), Cd(II) and Hg(II) from the aqueous environment.

**Table 3:** *R*^2^ and constant values for the different adsorption isotherm models of Zn(II), Cd(II) and Hg(II) on TLSB.

Materials	Parameters	Langmuir		Freundlich		Temkin		D–R	
Zn(II)	*R*^2^	0.9956		0.9586		0.8496		0.8995	
	Constants	*K*_*L*_	0.2130 L/mg	*K*_f_	47.53 L/g	*b*_t_	51.02 J/mol	*B*	0.029 × 10^–8^ mol^2^ J^2^
		*q*_max_	351.36 mg/g	1/*n*	0.1121	*a*_t_	2.15 × 10^10^ L/g	*ε*	8.58 kJ/mol
								*q*_max_	251.32 g/mg
Cd(II)	*R*^2^	0.9964		0.9662		0.9639		0.9096	
	Constants	*K*_L_	0.1744 L/mg	*K*_f_	20.09 L/g	*b*_t_	35.66 J/mol	*B*	0.102 × 10^–8^ mol^2^ J^2^
		*q*_max_	465.29 mg/g	1/*n*	0.2306	*a*_t_	1.73 × 10^10 ^L/g	*ε*	10.26 kJ/mol
								*q*_max_	308.09 g/mg
Hg(II)	*R*^2^	0.9950		0.8479		0.9089		0.4655	
	Constants	*K*_L_	0.1996 L/mg	*K*_f_	25.77 L/g	*b*_t_	39.07 J/mol	*B*	0.088 × 10^–8^ mol^2^ J^2^
		*q*_max_	211.02 mg/g	1/*n*	0.1881	*a*_t_	5.40 × 10^10^ L/g	*ε*	9.67 kJ/mol
								*q*_max_	311.55 g/mg

**Table 4:** Comparison of the adsorption capacities of various adsorbents.

Adsorbent	Adsorption capacity (mg/g)			References
	Zn(II)	Cd(II)	Hg(II)	
Guanyl-modified cellulose	78	68	48	^48^
Zn(II)-ionic imprinted polymer	20.833			^4^
Chitosan coated diatomaceous earth beads	127.4			^5^
Chitosan-based hydrogels		234.84		^65^
CPM		186.36		^73^
Fe_1−x_S NP/C microspheres			104	^11^
PAMAM-ATP			200.8	^72^
TLSB	357.29	458.32	208.12	This work

### Adsorption thermodynamics

To better understand the effect of temperature on adsorption, it is important to study thermodynamic parameters such as the standard Gibbs free energy change Δ*G*^0^, standard enthalpy Δ*H*^0^, and standard entropy Δ*S*^0^. The effect of temperature on adsorption processes is determined through the following relations (Eq. 12)^65,66^:

$$ \Delta G^{0} = \Delta H^{0} - T\Delta S^{0} $$

and the van’t Hoff equation, given as Eq. (13):

$$ \ln K = \frac{{\Delta S^{0} }}{R} - \frac{{\Delta H^{0} }}{RT} $$

where *K* (L/mol) is from the Langmuir equation and has units of liters per mole. *R* (8.314 J/mol K) is the universal gas constant, and *T* (K) is the adsorption temperature in Kelvin.

The plot of ln*K* against 1/*T* should be linear. The slope of the van’t Hoff plot is equal to − Δ*H*^0^/R, and its intercept is equal to Δ*S*^0^/R. The thermodynamic parameters obtained are listed in Table 5. The negative values of Δ*G*^0^ at different temperatures indicate the spontaneous nature of the adsorption process under the applied experimental conditions. The positive Δ*H*^0^ values confirm the endothermic behavior of TLSB. The positive values of Δ*S*^0^ may be attributed to the affinity of TLSB for zinc subgroup ions and increasing randomness at the solid–liquid interface. It can be concluded that adsorption by TLSB is an endothermic and spontaneous process based on the thermodynamic data of the adsorption isotherm. This conclusion was also confirmed as shown in Fig. 3c.

**Table 5:** Thermodynamic parameters for the adsorption of Zn(II), Cd(II), and Hg(II) on TLSB at different temperatures.

	Temperature/°C	Δ*G*^0^/(kJ/mol)	Δ*H*^0^/(kJ/mol)	Δ*S*^0^/(kJ/mol × K)
Zn(II)	20	− 0.986	+ 19.811	+ 0.053
	30	− 1.230		
	43	− 1.908		
	50	− 2.349		
Cd(II)	20	− 2.206	+ 14.716	+ 0.049
	30	− 2.513		
	40	− 2.897		
	50	− 3.265		
Hg(II)	20	− 0.763	+ 20.771	+ 0.077
	30	− 1.030		
	40	− 1.692		
	50	− 2.566		
	55	− 3.104		
	60	− 4.087		

## Desorption and regeneration studies

### Effect of various desorption reagents

**Figure 6:**
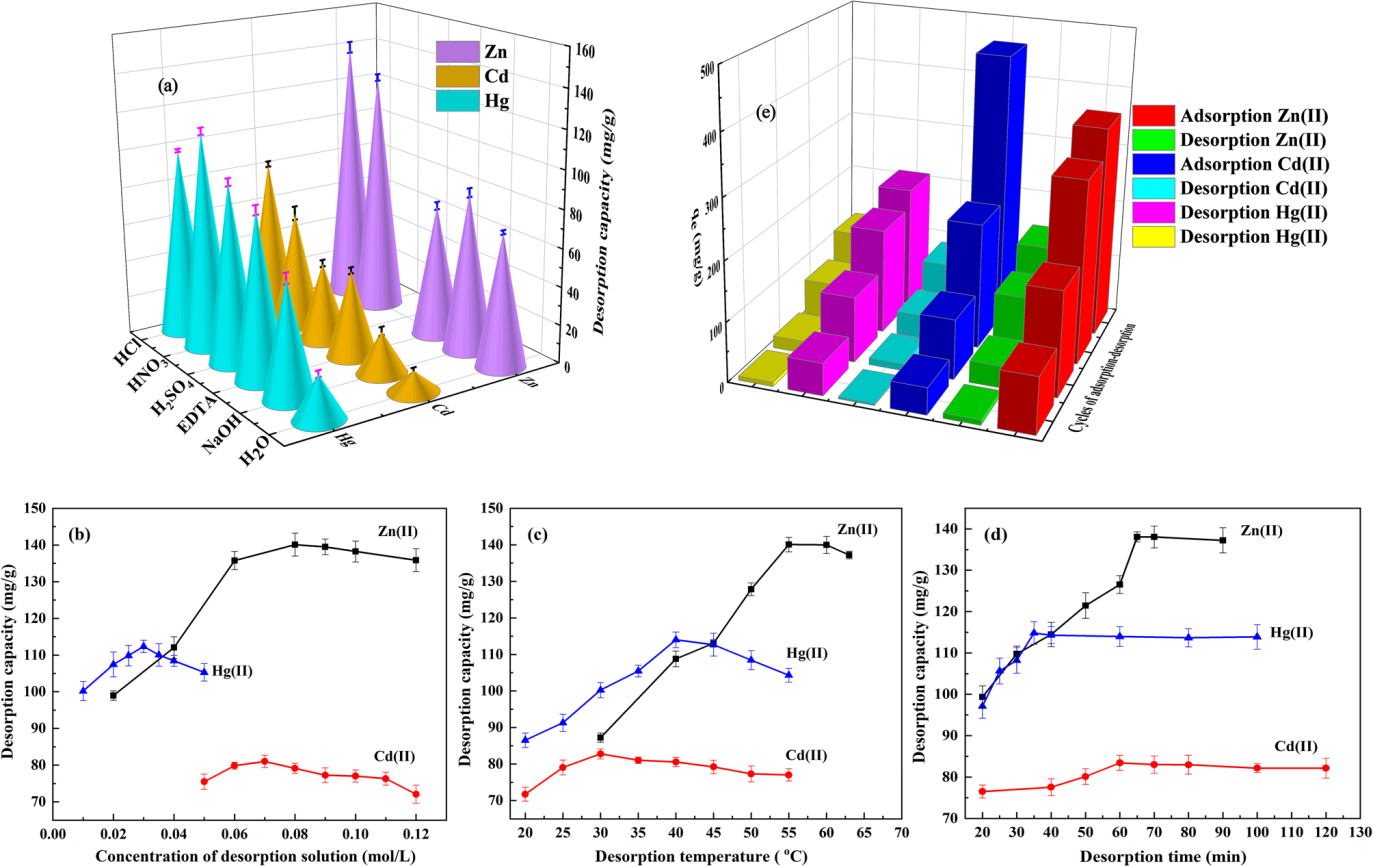
(**a**) Effect of various desorption reagents on the desorption capacity of TLSB for Zn(II), Cd(II) and Hg(II). (**b**) Effect of desorption reagent concentration on the desorption capacity of TLSB. (**c**) Effect of desorption temperature on the desorption capacity of TLSB. (**d**) Effect of desorption time on the desorption capacity of TLSB. (**e**) Adsorption/desorption cycles for Zn(II), Cd(II) and Hg(II) on TLSB.

Desorption reagents affect the desorption efficiency of TLSB. Out of various categories that encompass complexing agents and proton exchangers, six eluents were selected for testing. HCl, HNO_3_, H_3_PO_4_ and H_2_SO_4_ are regarded as proton exchangers. As complexing agents, NaOH and EDTA, a chelating agent, were chosen. The concentrations of the abovementioned six eluents were all set to 0.1 mol/L, and the effects of the various desorption reagents on the desorption capacity of TLSB are shown in Fig. 6a (Zn(II) desorption experiments: sample dosage 0.05 g, desorption temperature 56 °C, desorption time 65 min; Cd(II) desorption experiments: sample dosage 0.05 g, desorption temperature 30 °C, desorption time 60 min; Hg(II) desorption experiments: sample dosage 0.05 g, desorption temperature 40 °C, desorption time 35 min). The desorption process investigation revealed that the peak desorption of Zn(II) and Cd(II) from metal-loaded TLSB was achieved with HCl, while the maximum desorption of Hg(II) was achieved with HNO_3_. Hence, the desorption of Zn(II) and Cd(II) from adsorbents was performed using HCl, and Hg(II) was desorbed from the adsorbents using HNO_3_ in the following regeneration tests.

### Effect of desorption agent concentration

The effects of varying concentrations of desorption reagents on the desorption capacity of metal-loaded TLSB are presented in Fig. 6b (Zn(II) desorption experiments: sample dosage 0.05 g, eluent HCl, desorption temperature 56 °C, desorption time 65 min; Cd(II) desorption experiments: sample dosage 0.05 g, eluent HCl, desorption temperature 30 °C, desorption time 60 min; Hg(II) desorption experiments of: sample dosage 0.05 g, eluent HNO_3_, desorption temperature 40 °C, desorption time 35 min). The experimental results indicated that the amount of desorption increased with increasing desorption reagent concentration until it reached the maximum desorption capacity. This phenomenon could be elucidated on the grounds that the H^+^ concentration in the system was insufficient to completely replace the metal ions, causing the TLSB to lose its absorption properties. A high concentration of H^+^ ions can almost completely block the ions adsorbed onto TLSB, and the desorption capacity of metal-loaded TLSB could not increase further and even slightly decreased. The maximum desorption amount of metal-loaded TLSB reached 140.11 mg/g for Zn(II) and 81.02 mg/g for Cd(II) when the HCl concentration was 0.08 mol/L and 0.07 mol/L, respectively, and 112.36 mg/g for Ni(II) when the HNO_3_ concentration was 0.03 mol/L.

### Effect of desorption temperature

The effect of desorption temperature on the desorption capacity of metal-loaded TLSB is illustrated in Fig. 6c (Zn(II) desorption experiments: sample dosage 0.05 g, HCl concentration 0.08 mol/L, desorption time 65 min; Cd(II) desorption experiments: sample dosage 0.05 g, HCl concentration 0.07 mol/L, desorption time 60 min; Hg(II) desorption experiments: sample dosage 0.05 g, HNO_3_ concentration 0.03 mol/L, desorption time 35 min). H^+^ ions can compete with each other for active sites on the metal-loaded TLSB surface so that the desorption rate increases. When the temperature increased further, the desorption capacity of metal-loaded TLSB slightly decreased because the active sites could be weakened at higher temperature.

### Effect of sonication time

The effect of sonication time on the desorption capacity of metal-loaded TLSB is shown in Fig. 6d (Zn(II) desorption experiments: sample dosage 0.05 g, HCl concentration 0.08 mol/L, desorption temperature 56 °C; Cd(II) desorption experiments: sample dosage 0.05 g, HCl concentration 0.07 mol/L, desorption temperature 30 °C; Hg(II) desorption experiments: sample dosage 0.05 g, HNO_3_ concentration 0.03 mol/L, desorption temperature 40 °C). The desorption capacity increased with increasing sonication time, while the sonication time provided only a slight gain in the desorption capacity of metal-loaded TLSB. On the one hand, the agitation provided by ultrasound resulted in an enormous amount of heat being generated locally, and the desorption process was facilitated by a higher temperature. On the other hand, in pores, ultrasound can give rise to vortices that provide the heavy metal ions kinetic energy to escape from desorption sites; thus, the desorption capacity increased with increasing sonication time^67^.

### Regeneration and reuse

Recovering heavy metals adsorbed on the TLSB surface is significant for improving the economics of TLSB nanoadsorbents. To evaluate the ability to be reused of the TLSB adsorbent, adsorption/desorption cycles of TLSB were carried out. The adsorption and desorption cycles were replicated four times, as depicted in Fig. 6e. The adsorption capacity decreased gradually with increasing cycles. The decreasing adsorption capacity during regeneration cycles could be explained by the nanostructure of TLSB and the destruction of its adsorption active sites at low pH values. The results showed that TLSB could be recycled up to three times for Zn(II), three times for Cd(II) and two times for Hg(II) ions with very little efficiency loss.

## Adsorption mechanism

**Figure 7:**
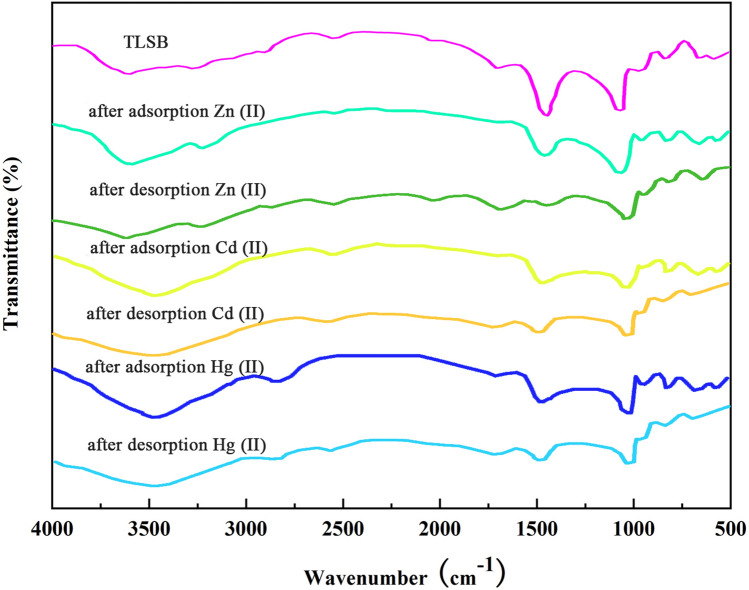
FTIR spectra of TLSB and samples after the adsorption and desorption of Zn(II), Cd(II) and Hg(II).

To clarify the adsorption mechanisms of Zn(II), Cd(II) and Hg(II) on TLSB, FTIR analysis of TLSB, metal-loaded TLSB and desorbed Zn(II), Cd(II), Hg(II) samples within the wavenumber range of 500–4000 cm^−1^ was performed. As shown in Fig. 7, the absorbance bands of TLSB were found at 3400–3690 cm^−1^, with a peak at 3481 cm^−1^, which can be attributed to the O–H stretching of hydroxyl groups and intermolecular hydrogen bonds. This peak shifted to lower wavenumbers of 3620 cm^−1^, 3486 cm^−1^, and 3521 cm^−1^ and weakened after adsorption, indicating that the O–H and hydrogen bonds of TLSB were broken, which in turn demonstrates the involvement of oxygen in the chemisorption of Zn(II), Cd(II) and Hg(II) and subsequent complexation process^68^. The bands at 2864 and 2574 cm^−1^ were related to the C–H stretching of the C–S skeleton and S–H and nearly disappeared after adsorption, indicating that the thiol groups of TLSB were complexed with ions, and these peaks appeared after desorption. The characteristic absorption peak at approximately 1718 cm^−1^ revealed C=O stretching in carboxylic organic acid groups, which shifted to lower wavenumbers after adsorption and appeared at 1713 cm^−1^, 1719 cm^−1^, and 1720 cm^−1^ after desorption. The S–H bond characteristic absorption peak at 985 cm^−1^, which weakened and shifted to 973 cm^−1^, 960 cm^−1^, and 957 cm^−1^, respectively, after adsorption, may be explained by the thiol groups of TLSB forming complexes with heavy metal ions. In addition, the FTIR spectrum of TLSB after desorption of ions was almost the same as that of the original TLSB, which indicated that TLSB can be reused. These changes in the absorption peaks indicated that the activated sites on the surface of TLSB contained hydroxyl, carboxylic and thiol functional groups, which formed new chemical bonds with Zn(II), Cd(II), and Hg(II). Furthermore, ion exchange and electrostatic attraction may also occur in the adsorption process, yet chemical adsorption is the main adsorption process^69^. More importantly, during the process of metal adsorption and desorption, the pore structures and properties of TLSB remained relatively stable, indicating that TLSB has great potential for applications in the removal of the zinc subgroup ions Zn(II), Cd(II), and Hg(II) from water.

**Figure 8:**
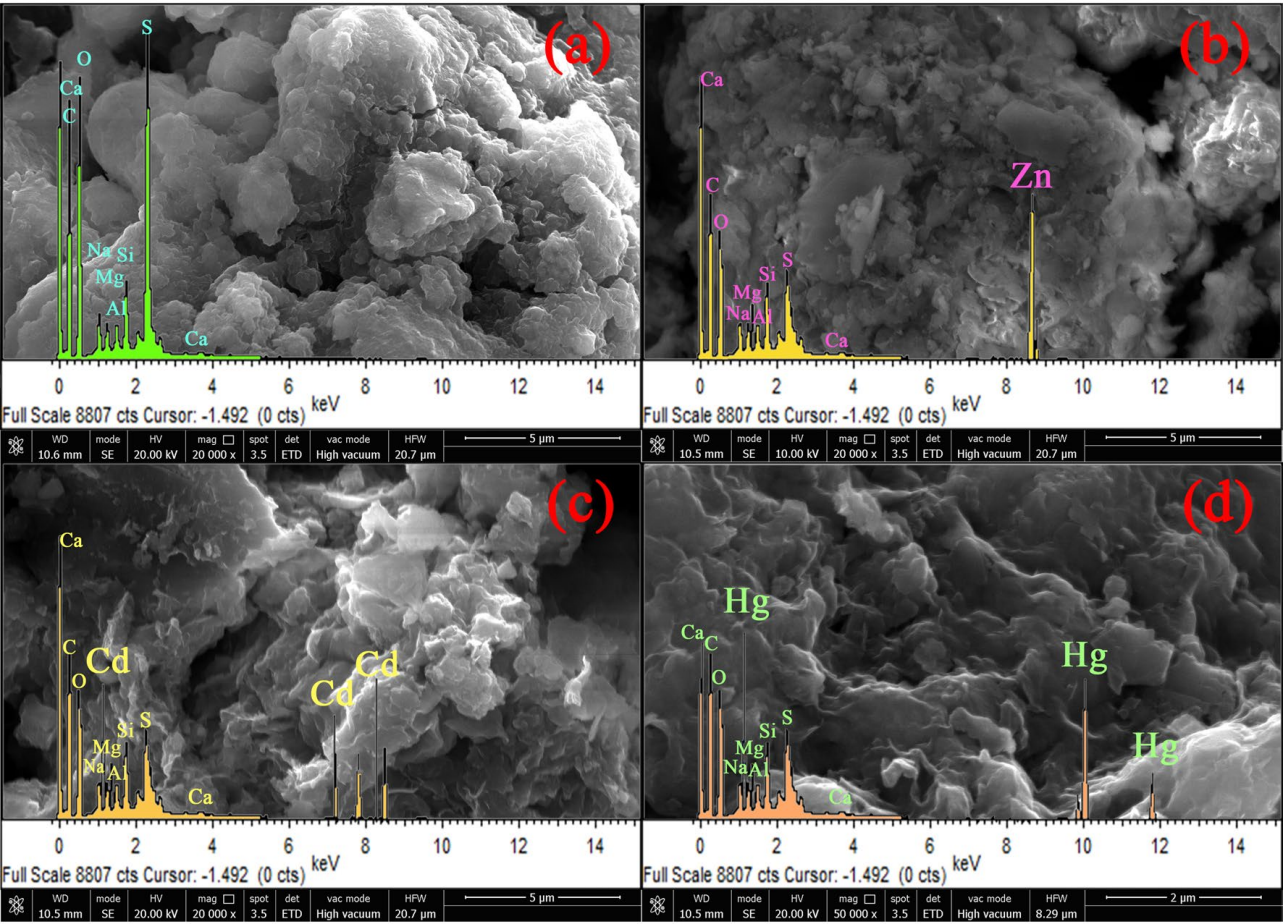
SEM–EDX images of TLSB (**a**) and samples after the adsorption of Zn(II) (**b**) Cd(II) (**c**) and (**d**) Hg(II).

To further elucidate the adsorption mechanism of Zn(II), Cd(II) and Hg(II) onto TLSB, samples were analyzed by using SEM and EDX. SEM micrographs of TLSB and metal-loaded TLSB are shown in Fig. 8a,d. The surface morphology of TLSB was considerably changed after adsorption. The image of TLSB in Fig. 8a shows that there are irregular shapes and sizes with steps, bends and broken edges on the surface of TLSB, and this encourages metal ions to diffuse from the outside to the inside of the material, leading to a greater specific surface area and more active sites for the metal ion desorption process^70^. SEM images after adsorption are given in Fig. 8b–d. It is observed that the mentioned structure on the TLSB surface was destroyed. The additional bright spots on the metal-loaded TLSB are adsorbed ions, which were verified by EDX analysis. It can also be observed that the distribution of bright spots is not uniform, indicating that only specific functional groups are involved in the adsorption process of ions^71^. EDX analysis was also conducted to evaluate the adsorption on TLSB (Fig. 8a,d). The EDX spectrum of TLSB indicated the presence of C, O, S, Na, Ca, Mg, Al and Si in the structure (Fig. 8a) but did not exhibit the characteristic signal of ions on the surface of TLSB. In the EDX spectra of TLSB after adsorption experiments (Fig. 8b–d), new characteristic peaks of Zn(II), Cd(II) and Hg(II) emerged and accompanied the decreases in the peaks of C, O, S, Na, Ca, Mg, Al and Si, which should be attributed to chemical adsorption and ion exchange^72^. To summarize, these results show that the adsorption of TLSB is probably a chemical adsorption mechanism, which lends credence to the adsorption mechanism that was postulated earlier.

## Conclusions

In this work, TLSB was prepared and investigated for removing the zinc subgroup ions Zn(II), Cd(II) and Hg(II) from aqueous solution. The optimum conditions of adsorption for TLSB occur at pH 4.84, 4.52 and 5.18, with adsorption temperatures of 43 °C, 40 °C and 55 °C and contact times of 100 min, 40 min, and 105 min, respectively. The maximum adsorption capacity of TLSB was 357.29 mg/g, 458.32 mg/g and 208.12 mg/g, respectively. The adsorption kinetics and isotherms showed that the adsorption processes were best described by the pseudo-second-order and Langmuir models. Desorption experiments revealed that HCl was able to elute 140.30 mg/g Zn(II) and 83.44 mg/g Cd(II), and HNO_3_ was able to elute 114.85 mg/g Hg(II). The regeneration experiments demonstrated that the adsorption/desorption capacity of TLSB remained at a relatively high level after 2–3 rounds of recycling. Furthermore, the FTIR and SEM–EDX results demonstrated that zinc subgroup ions were mainly adsorbed by selected activated groups in coarse mesopores on the surface of the TLSB nanocomposite, and the adsorption mechanism was likely a chemisorption process. In addition, the ability of the metals to bind with TLSB decreased in the following order: Cd(II) > Zn(II) > Hg(II). Hence, the TLSB nanocomposite was confirmed to be a sustainable and efficient adsorbent to remove Zn(II), Cd(II) and Hg(II) from aqueous environments.

## Materials and methods

### Materials

Lignocellulose was procured from Beijing Ming Ang Rui Xiang Technology Co., Ltd., Beijing, China. For sodium bentonite, the cationic exchange capacity (CEC) was 90 mol/100 g, 100 mol/100 g and 110 mol/100 g (purchased from Zhejiang Feng Hong Clay Chemical Co., Huzhou, China). Thioglycolic acid was of guaranteed reagent grade and was obtained from Tianjin Damao Chemical Co., Tianjin, China.

### Preparation of thiol-lignocellulose (TL)

Thioglycolic acid (53.20 g) and acetic anhydride (25.92 g) were mixed thoroughly, cooled to room temperature, added to lignocellulose powder (10.00 g), and stirred in a 40 °C thermostatic water bath for 48 h. The mixture was then suctioned, and the dark-green precipitate was cleansed with deionized water for a few rounds until it reached pH 7.00 (PB-10, Shanghai, China) and subsequently filtered. The above product was soaked and stirred for 10 h with 2% NaOH, and the dark-green solution produced was filtered. Then, the precipitate was stirred in 2% HCl for 2 h, and the solution became a gray-green flocculent suspension. After standing and settling, the precipitate (gray-green) was centrifuged (H2050R, Hunan, China), cleansed with deionized water several times, and subsequently vacuum-dried for 10 h at 48 °C to achieve a yellow-green powdery TL product. The obtained products were stored dry.

### Preparation of thiol-lignocellulose/sodium bentonite (TLSB)

A homogenous TL suspension was prepared by dissolving TL in a certain concentration of NaOH solution [the ratio between the weight of TL (g) and the volume of NaOH (mL) was 1:30] in batches and stirring for 40 min (SHA-C, Jiangsu, China). Then, an equal amount of SB (with respect to the weight of TL) was added to a certain volume of deionized water (the ratio between the weight of SB (g) and the volume of deionized water (mL) was 1:30) and stirred for 30 min, forming a uniform SB suspension^21^. After that, the SB suspension was slowly added to the TL suspension at a certain temperature and stirred for several hours. The reaction mixture was centrifuged and washed with deionized water to pH 7.00. The precipitate was dehydrated under vacuum at 70 °C for 10 h to obtain a gray-yellow powdery TLSB nanocomposite.

### Standard working curves of Zn(II), Cd(II) and Hg(II) ions

A series of standard solutions were poured into a 1-cm-deep quartz-colored dish. The desorption was scanned at wavelengths of approximately 570 nm, 620 nm and 610 nm using a double beam ultraviolet (UV)-visible spectrophotometer (TU-1901, Beijing, China). Taking the concentration *C* as the abscissa and the absorbance (Abs) as the ordinate, measured values were can be used to obtain standard working curves for the sample liquid, and pertinent coefficients were calculated to estimate the standard working curve's pertinence. The standard working curve equation for Zn(II) was *y* = 0.03297*x* + 0.401 (Eq. a) and the curve’s correlation coefficient was *R*^2^ = 0.9998. The standard working curve equation for Cd(II) was *y* = 0.5903*x* + 3.6218 (Eq. b), and the curve’s correlation coefficient was *R*^2^ = 0.9991. The standard working curve equation for Hg(II) was *y* = 0.0272*x* + 1.7067 (Eq. c), and the curve’s correlation coefficient was *R*^2^ = 0.9991. (The details of the experimental procedure used to obtain the standard working curves of ions are provided in the Supporting Information).

### Adsorption, desorption and regeneration experiments

TLSB (0.05 g) was dispensed into 50 mL of aqueous Zn(II), Cd(II), and Hg(II) solutions in 100 mL Erlenmeyer flasks, and the flasks were placed on a shaker set at 120 rpm for a while. The absorbance of heavy metal complexes was studied in single-component experiments with 3 different solutions: sodium acetate/glacial acetic acid (NaOAc/HOAc) buffer solution and xylenol orange (XO); hexamethylenamine buffer solution and XO; and NaOAc/HOAc buffer solution and iodide-ascorbic-acid-Rhodamine B. Samples were tested for adsorption in a double beam ultraviolet (UV)-visible spectrophotometer, with the absorbance values at wavelengths of approximately 570 nm, 620 nm and 610 nm. Then, the residual metal ion concentrations were determined with linear regression equations (Eq. a–c) for Zn(II), Cd(II), and Hg(II). The adsorption experiments were carried out by using different concentrations of metal ions, pH values, adsorption temperatures, and adsorption times of heavy metal ions. Each monocomponent adsorption experiment was conducted thrice, and the results are provided as average values. The adsorption capacity of each heavy metal solution was determined from the following equation^73^:

14 $$ q_{t,1} = \frac{{(C_{0} - C_{t,1} )V_{1} }}{{m_{1} }} $$

where *q*_t,1_ (mg/g) is the adsorption capacity of the heavy metal ions at time *t* (min); *C*_0_ and *C*_t,1_ (mg/L) refer to the initial concentrations and the final concentration at time *t* (min); *V*_1_ (mL) refers to the volumes of the Zn(II), Cd(II), and Hg(II) solutions; and *m*_1_ (g) is the mass of TLSB.

A total of 0.05 g metal-loaded TLSB nanocomposite was transferred into 50 mL of various desorption agents, and then the systems were vigorously mixed under ultrasonic dispersion (KS-300EI, Ninbo, China) at 100 W and 40 kHz at a given temperature until each monocomponent system reached desorption equilibrium. The suspensions were centrifuged, and the metal ion concentration in the supernatant from each desorption experiment was tested by the method described in the adsorption experiments. The desorption experiments were conducted thrice in the same manner, and the desorption capacity at equilibrium (*q*_*t,2*_) was calculated using the equation given below^74^:

15 $$ q_{t,2} = \frac{{C_{t,2} V_{2} }}{{m_{2} }} $$

where *q*_t,2_ (mg/g) is the amount at desorption time *t* (min); *C*_t,2_ (mg/L) is the concentration of each metal ion in solution at time *t* (min); *V*_2_ (mL) is the volume of the desorption agent; and *m*_2_ (g) is the final mass of the TLSB after the metal ions were released.

To examine the ability of the TLSB nanocomposite to be reused, adsorption/desorption cycle testing was repeated under the optimum conditions. After the first batch of monocomponent adsorption experiments, the TLSB was cleansed using deionized water until the pH value was near neutral before performing the subsequent adsorption experiments. The regenerated TLSB was used in four consecutive cycles under identical conditions, and the Zn(II), Cd(II) and Hg(II) residual concentrations in solution were measured by the method mentioned above for every adsorption/desorption cycle experiment, and the adsorption/desorption capacity was calculated by the equations mentioned above.

## Supplementary information

Supplementary Information.
