# Supplementary material for: Enhanced heavy metal removal from an aqueous environment using an eco-friendly and sustainable adsorbent
Source: Sci Rep. 2020 Oct 5;10:16453. doi: 10.1038/s41598-020-73570-7 (PMC7536411; doi:10.1038/s41598-020-73570-7)
Supplement: Supplementary file 1 — Supplementary Information. [file 41598_2020_73570_MOESM1_ESM.pdf]

Supporting Information

Enhanced Heavy Metal Removal from an Aqueous Environment Using an Eco-Friendly and Sustainable Adsorbent

Wanqi Zhang[1,†], Yuhong An[1,†], Shujing Li[1], Zhechen Liu[1], Zhangjing Chen[2], Yukun Ren[3], Sunguo Wang[4], Xiaotao Zhang[5,6,*], Ximing Wang[1,6,*]

[1] College of Material Science and Art Design, Inner Mongolia Agricultural University, Hohhot, China.
[2] Department of Sustainable Biomaterials, Virginia Polytechnic Institute and State University, Blacksburg, VA, USA.
[3] Bioimaging Research, Sanofi Global R&D, Framingham, MA, USA.
[4] Sungro Bioresource & Bioenergy Technologies Corp, Alberta, Canada.
[5] College of Science, Inner Mongolia Agricultural University, Hohhot, China.
[6] Inner Mongolia Key Laboratory of Sandy Shrubs Fibrosis and Energy Development and Utilization, Hohhot, China.
*Correspondence should be addressed to Xiaotao Zhang and Ximing Wang.
Email: lianzixiaotao@163.com; wangximing@imau.edu.cn.
[†]Contributed equally to this paper.

1. Standard Work Curve of Zn(II) Ions

Zn(II) standard solution was prepared by dissolving 1.2520g ZnO in deionized water with addition of 10mL concentrated sulfuric acid. Xylenol orange (XO) standard solution was prepared by dissolving 0.15000g xylenol orange in deionized water in a volumetric flask (100mL). Glacial acetic acid solution was prepared by dissolving 36.0mL glacial acetic acid in deionized water in a volumetric (100mL). Sodium acetate/glacial acetic acid (NaOAc/HOAc) buffer solution was prepared by dissolving 200.0g sodium acetate in deionized water with addition of 26.0mL glacial acetic acid solution in a volumetric flask (1L).

In order to prepare calibration curve the following volumes of Zn(II) working standard solution 2.5mL, 5.0mL, 7.5mL, 10.0mL, 12.5mL, 15.0mL, 17.5mL, 20.0mL, 22.5mL and 25.0mL were placed in ten clean volumetric flask(50mL). Then, 10.0mL Sodium acetate/glacial acetic acid (NaOAc/HOAc) buffer solution and 2.5mL Xylenol orange (XO) standard solution were added to each flask, respectively. And then add deionized water to scale. The solution was stable for 10 min. A series of samples of Zn(II) were transferred into the 1cm of quartz color dish and scanned absorption in a double beam ultraviolet (UV)-visible spectrophotometer (TU-1901, Beijing Purkinje General Instrument Co., Ltd., Beijing, China) at the wavelength about 570nm with water as reference. As shown in Figure S1,Taking the concentration C(mg/L) of Zn(II) as the abscissa and the absorbance (Abs) as the ordinate, the arithmetic of one unit linearity return can be used to obtain the standard working curve of the sample liquid and pertinent coefficient was calculated to estimate standard working curve's pertinence.

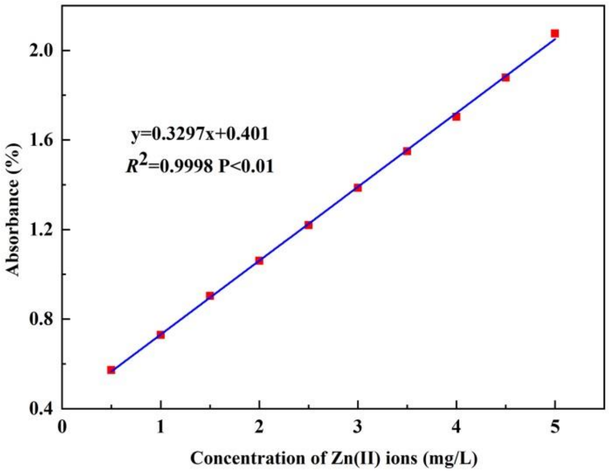

Figure S1. Zn(II) standard work curve.

2. Standard Work Curve of Cd(II) Ions

Cd(II) standard solution with accuracy of 2.0mL were put into a volumetric flask(50mL).Then,4.0mL 1mol/L sulfuric acid, 1 mL 20%KI-2%ascorbic solution, 10.0mL 1%PVA-124 solution and 3.0mL 0.05% Rhodamine B were added to the flask. And then add deionized water to the scale. The solution was stable for 5 min. The sample of Cd(II) were transferred into the 1cm of quartz color dish and scanned absorption in a double beam ultraviolet (UV)-visible spectrophotometer (TU-1901, Beijing Purkinje General Instrument Co., Ltd., Beijing, China) at the wavelength about 620 nm with water as reference.

In order to prepare calibration curve the following volumes of Cd(II) working standard solution 1.0mL, 2.0mL, 4.0mL, 6.0mL, 8.0mL, 10.0mL were placed in ten six volumetric flask(50mL). Then,4.0mL 1mol/L sulfuric acid, 14mL 20% KI-2% ascorbic solution, 10.0mL 1%PVA-124 solution and 3.0mL 0.05% Rhodamine B were added to the flask. And then add deionized water to the scale. The solution was stable for 5 min. A series of samples of Cd(II) were transferred into the 1cm of quartz color dish and scanned absorption in a double beam ultraviolet (UV)-visible spectrophotometer (TU-1901, Beijing Purkinje General Instrument Co., Ltd., Beijing, China) at the wavelength about 620nm with water as reference. As shown

in Figure S2,Taking the concentration C(μg/L) of Cd(II) as the abscissa and the absorbance (Abs) as the ordinate, the arithmetic of one unit linearity return can be used to obtain the standard working curve of the sample liquid and pertinent coefficient was calculated to estimate standard working curve's pertinence.

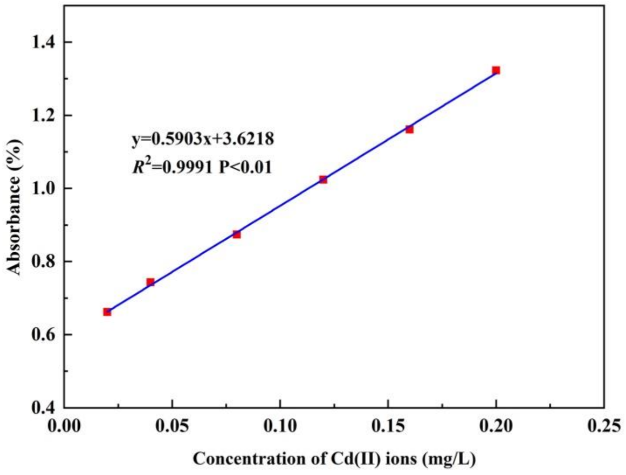

Figure S2. **Cd(II) standard work curve.**

3. Standard Work Curve of Hg(II) Ions

Hg(II) standard solution was prepared by dissolving 0.3426g $Hg(NO_3)_2$-H_2O in deionized water with addition of 1 mL HNO_3 in a volumetric flask (100mL). Rhodamine B solution was prepared by dissolving 0.100g Rhodamine B in deionized water in a volumetric flask (100mL). 20%KI-2%ascorbic solution was prepared by dissolving 50gKI and 5g ascorbic in deionized water in a volumetric flask (250mL). PVA-124 solution was prepared by dissolving 5g polyvinyl alcohol in in deionized water in a volumetric flask (100mL). 10g sodium acetate was dissolved in deionized water, and then added glacial acetic acid until the pH of the solution reached 3.19.

In order to prepare calibration curve the following volumes of Hg(II) working standard solution 0.5mL, 1.0mL, 1.5mL, 2.0mL, 2.5mL and 3.0mL were placed in six clean volumetric flask(25mL).Then, 4mL sodium acetate/glacial acetic acid (NaOAc/HOAc) buffer

solution, 4mL 20%KI-2%ascorbic solution, 1.5mL 0.1% rhodamine B and 1.75mL 5% PVA-124 solution was added to each flask, respectively. And then add deionized water to scale. A series of samples of Hg(II) were transferred into the 1cm of quartz color dish and scanned absorption in a double beam ultraviolet (UV)-visible spectrophotometer (TU-1901, Beijing Purkinje General Instrument Co., Ltd., Beijing, China) at the wavelength about 610 nm with water as reference. As shown in Figure S3,Taking the concentration C(μg/L) of Hg(II) as the abscissa and the absorbance (Abs) as the ordinate, the arithmetic of one unit linearity return can be used to obtain the standard working curve of the sample liquid and pertinent coefficient was calculated to estimate standard working curve's pertinence.

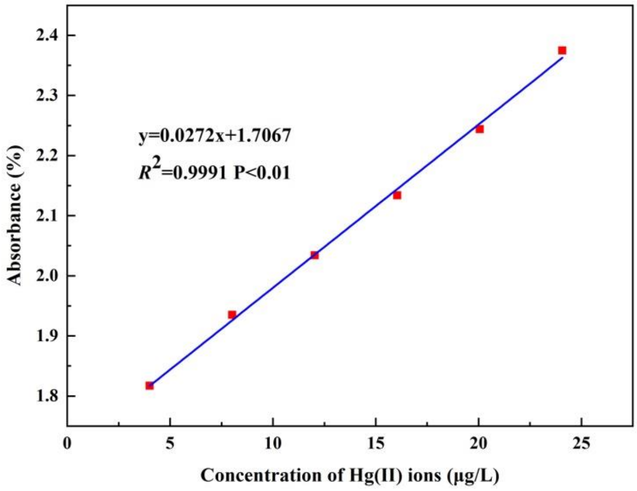

Figure S3. **Hg(II) standard work curve.**
